# Comparing leukocyte‐ and thrombocyte‐rich fibrin and autologous grafts for the treatment of experimentally induced wounds in the tarsometatarsus of chickens

**DOI:** 10.1002/vetr.70220

**Published:** 2025-12-26

**Authors:** Vivian Ferreira Rech, Sheila Canevese Rahal, Renata Dalcol Mazaro, Luna Scarpari Rolim, Guilherme Rech Cassanego, Matheus Yuri Dos Santos, Rafael Almeida Fighera

**Affiliations:** ^1^ Department of Veterinary Surgery and Animal Reproduction, School of Veterinary Medicine and Animal Science São Paulo State University (UNESP) Botucatu Brazil; ^2^ Veterinary Pathology Laboratory, Department of Pathology Federal University of Santa Maria (UFSM) Santa Maria Brazil

**Keywords:** autologous, bird, bone exposure, fibrin, skin defect

## Abstract

**Background:**

Managing open wounds in birds is challenging. This study compared leukocyte‐ and thrombocyte‐rich fibrin (L‐TRF) and autologous grafts for treating wounds in the tarsometatarsus of chickens.

**Methods:**

Full‐thickness wounds were experimentally induced in the tarsometatarsus of 23 healthy adult chickens. The wounds were then treated with either a bandage dressing (G1, *n* = 7), an L‐TRF membrane (G2, *n* = 8) or an autologous skin graft (G3, *n* = 8). Wounds were assessed on days 7, 14, 21 and 28. In G2, L‐TRF membranes were reapplied at each time point.

**Results:**

By day 28, 71.4% of wounds in G1 remained unhealed, while 87.5% in G2 were fully healed. Wound reduction was significantly greater in G2 than G1 at all times, especially on days 14 and 21. No difference in wound area was observed on day 7, but wounds in G2 were smaller than in G1 on days 14, 21 and 28. By day 28, 75% of G3 grafts survived, while 25% showed necrosis. Histology showed higher angiogenesis and collagen production in G2 than G3, with more inflammation in G3. Re‐epithelialisation was higher in G2 than in G1.

**Limitations:**

Wound healing in the tarsometatarsus resulted in tissue differing from the original, regardless of the treatment applied.

**Conclusions:**

The L‐TRF membrane was the most effective treatment for skin wounds induced in the tarsometatarsus of chickens, outperforming autologous skin grafts.

## INTRODUCTION

The skin of birds is highly susceptible to injury due to its anatomical characteristics, such as thin and delicate thickness.^1^ Managing large open wounds in birds is frequently challenging.^2^ Suturing cutaneous defects in the distal portions of the pelvic limbs is particularly complex, given the lack of loose skin and the risk of restricted limb movement due to wound contracture overlying the joints.^2^, ^3^ Additionally, skin wounds in the tarsometatarsus region may expose bone due to poor muscle coverage.^4^

Skin wound treatment in birds generally follows techniques and principles used in mammals but requires some adjustments due to their unique characteristics.^1^ The choice of treatment depends on the wound's specific features but must also consider factors such as immobilisation time, stress, frequency of bandage changes and overall treatment duration.^5^

Platelet‐rich fibrin (PRF) is a platelet concentrate obtained through blood centrifugation without the use of anticoagulants.^6^, ^7^, ^8^ PRF forms a fibrin matrix that contains platelets, leukocytes, cytokines and circulating stem cells.^7^ Autologous PRF has shown positive results in the treatment of naturally occurring skin wounds in dogs^8^ and cats.^9^ It has also proven effective in experimentally induced skin wounds in cats, promoting collagen production through growth factors and reducing inflammation.^10^ Additionally, one study used canine‐derived PRF to treat wounds in cats.^11^ The synergistic effects of growth factors and fibroblast recruitment facilitate collagen synthesis and tissue regeneration.^12^ Although the production of leukocyte‐ and thrombocyte‐rich fibrin (L‐TRF) membranes has been studied in birds,^13^ research on their application in avian skin wound treatment remains limited. Studying L‐TRF in birds offers insight into wound‐healing mechanisms in a species with distinct integumentary features, while also enhancing the translational relevance of these therapies through comparative applications in mammalian models.

Autologous skin grafting involves the removal of a portion of the dermis and epidermis from one site and its transfer to a recipient bed on the same animal.^2^, ^3^, ^14^ This technique is commonly used in mammalian medicine to treat extensive skin defects but has been less frequently explored in birds.^2^, ^15^, ^16^, ^17^ Potential benefits of skin grafting in avians include a faster return to function and reduced treatment duration compared to topical therapies and dressings.^15^, ^17^, ^18^ Moreover, birds possess a thin dermal layer that may facilitate rapid revascularisation, potentially contributing to the success of skin grafts.^19^

To develop more suitable protocols for treating skin wounds with tissue loss and bone exposure in birds, this study compared the L‐TRF and autologous grafts in the treatment of skin defects induced in the tarsometatarsus, using chickens as an experimental model. The hypothesis was that L‐TRF treatment would protect the wound and stimulate faster healing compared to autologous grafts.

## METHODS

### Animal selection

Twenty‐three adult, healthy chickens (*Gallus gallus domesticus*), weighing between 1.2 and 1.6 kg, were used. The chickens were housed in a closed space (3.3 m wide × 3.3 m long × 3 m high) with ventilation and were fed a commercial diet with water provided ad libitum. The chickens were randomly divided into three groups using GraphPad software (QuickCalcs Randomisation): group 1—control (*n* = 7), group 2—L‐TRF membrane (*n* = 8) and group 3—autologous skin graft (*n* = 8).

### Surgical procedure and wound management

The chickens were moved to a temperature‐controlled surgical centre for thermal comfort. Tranquilisation was achieved with 10 mg/kg ketamine (Cetamin) and 2 mg/kg xylazine (Xilazin), administered intramuscularly (IM), while 2 mg/kg morphine (IM) (DIMorf) was included for its analgesic effect. After 10 minutes, general anaesthesia was induced with isoflurane (Isoforine) via a face mask and subsequently maintained through a non‐cuffed endotracheal tube.

**FIGURE 1:**
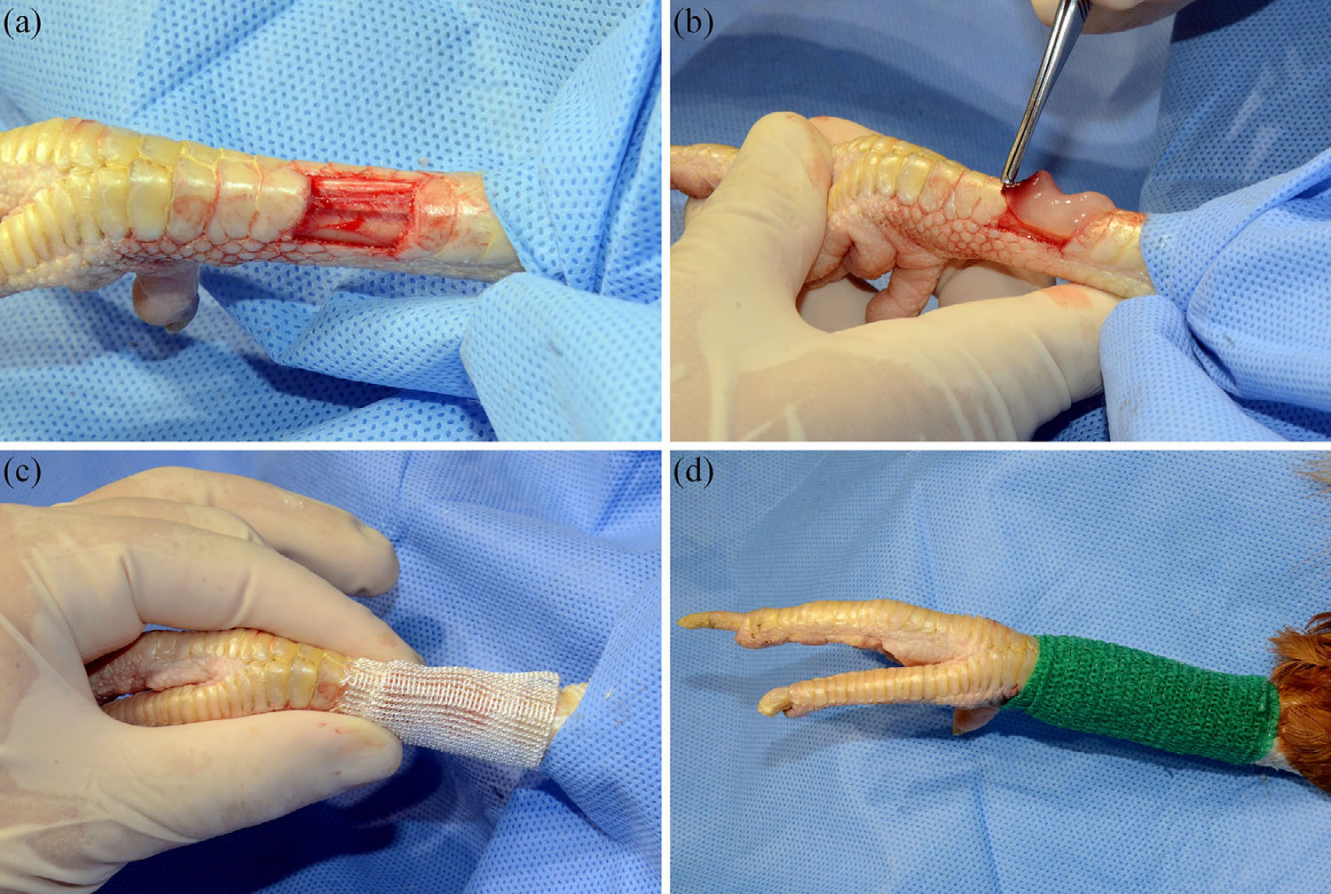
(a) Wound skin induced on the tarsometatarsus of a chicken showing bone exposure. (b) Application of a leukocyte‐ and thrombocyte‐rich fibrin membrane to the wound bed (group 2). (c) First layer of dressing with a Rayon bandage. (d) Second layer of dressing with an elastic bandage.

The birds were placed in dorsal recumbency, and antisepsis of the tarsometatarsus area was performed using chlorhexidine. A 2 cm × 1 cm wound was created on the medial surface of the tarsometatarsus using a scalpel to delineate the flap. The flap was then elevated, including the skin, subcutaneous tissue and periosteum, thereby exposing the osseous surface of the tarsometatarsus (Figure 1a).

In group 1, the skin wound received only a dressing, which consisted of a Rayon bandage, an adhesive elastic bandage and adhesive tape to secure the ends.

In group 2, 2.5 mL of blood was collected from the right jugular vein while the birds were manually restrained. The blood was collected using a 5 mL syringe and a 30 × 0.8 mm needle, and immediately transferred to a sterile plastic tube without additives. The tube was centrifuged at 1500 rpm for 15 minutes using an analog centrifuge (model 80‐2B, 4000 rpm). After centrifugation, the blood separated into layers: platelet‐poor plasma in the supernatant, L‐TRF clot in the middle and red blood cells at the base. The supernatant was discarded, and the L‐TRF clot was carefully withdrawn from the tube. The red blood cell layer was gently separated with a spatula to preserve the buffy coat. The L‐TRF clot was then placed in a PRF Box (FibrinBOX) to form a membrane. For this purpose, the L‐TRF clot was positioned on the grid of the PRF Box and pressed with a specialised metal plate. The membrane was applied over the wound bed (tarsometatarsus defect), extending beyond the wound margins (Figure 1b). No sutures were used to secure the membrane. A dressing like that used in group 1 was then applied (Figure 1c,d). The bandage dressings were of different colours to distinguish the groups.

For harvesting the full‐thickness skin graft in group 3, the inguinal region was prepared by plucking feathers and performing aseptic cleaning with chlorhexidine. A skin fragment measuring 2 cm in height and 1 cm in width was excised. The donor site was closed with 3‐0 monofilament nylon using a simple continuous suture pattern. The skin fragment was held between the index finger and thumb, and a scalpel blade was used to remove the subcutaneous tissue until the feather follicles were identified. Five full‐thickness incisions, approximately 3 mm in length, were made through the graft using a scalpel blade. The graft was then sutured to the wound edges using a simple interrupted pattern with 4‐0 monofilament nylon. Before applying the same dressing used in the other groups, a thin layer of neomycin sulphate and bacitracin dermatological ointment was applied to reduce adherence between the graft and the dressing, rather than for its antibiotic effect.

Following induction of anaesthesia, all birds received 10 mg/kg enrofloxacin and 0.5 mg/kg meloxicam (IM). Enrofloxacin every 24 hours was continued for 7 days, while meloxicam was administered orally every 24 hours for 3 days. Additionally, 25 mg/kg dipyrone (IM) was administered every 12 hours for 5 days to provide analgesia. The chickens were monitored daily during the postoperative period. Dressing changes and L‐TRF membrane replacements were performed every 7 days.

**FIGURE 2:**
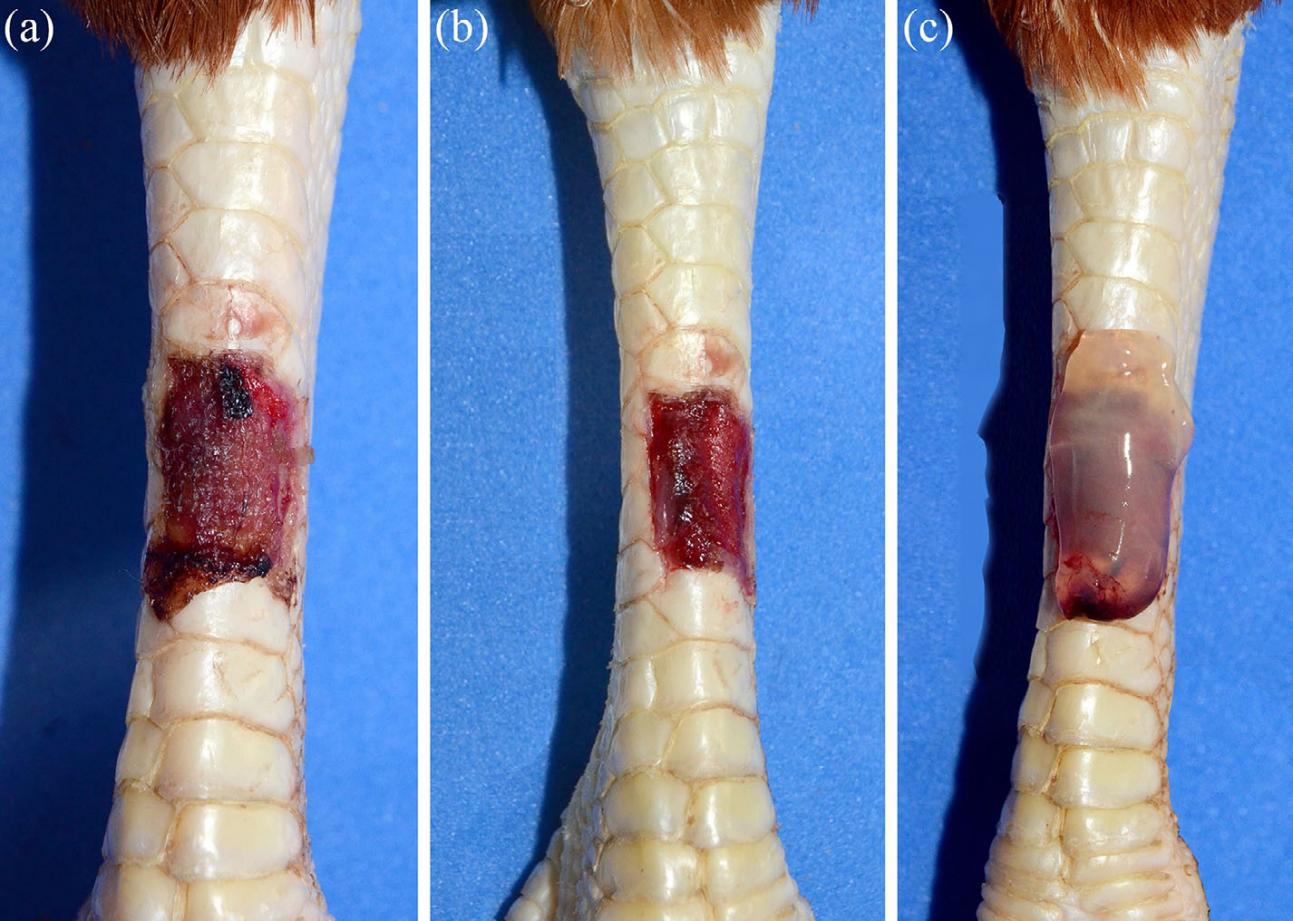
Wound skin induced on the tarsometatarsus of a chicken treated with leukocyte‐ and thrombocyte‐rich fibrin (L‐TRF) membrane (group 2). (a) Appearance of the wound with the L‐TRF membrane seven days postoperatively. (b) Wound after removal of the L‐TRF membrane. (c) Application of a new L‐TRF membrane.

In groups 1 and 2, the wounds were irrigated with 0.9% saline solution before removing the Rayon bandage. In group 2, after removing the L‐TRF membrane, the wound was irrigated with 0.9% saline solution, and a new L‐TRF membrane was applied (Figure 2). In group 3, precautions were taken to prevent graft displacement, and a thin layer of neomycin sulphate and bacitracin ointment was applied before placing a new dressing.

Each skin wound was photographed on postoperative days 7, 14, 21 and 28. The birds were positioned consistently, with the wound placed 20 cm from the camera (Nikon D7000, 18–55 mm lens). A ruler was positioned adjacent to the wound for scale reference.

Wounds in groups 1 and 2 were measured using ImageJ software (Fiji, version 2.14.0). Pixels were converted to millimeters using the scale ruler visible in the image. The wound area was delineated by tracing its perimeter with the freehand tracing tool. In group 3, graft images were analysed for nonviable areas, which were characterised by white or black discolouration, elevation from the graft bed or signs of necrosis.

### Euthanasia and histological processing

Four weeks after surgery, the birds were euthanased to collect lesions for histological analysis. The birds were sedated with 30 mg/kg ketamine hydrochloride, 4 mg/kg xylazine hydrochloride, 2 mg/kg midazolam and 2 mg/kg morphine administered IM. Midazolam and morphine were administered to provide additional sedation, muscle relaxation and analgesia, ensuring a smooth and humane induction of anaesthesia prior to euthanasia. Subsequently, propofol (10 mg/kg) was administered intravenously to achieve a deep plane of anaesthesia, followed by potassium chloride until cardiorespiratory arrest occurred.

The collected specimens were fixed in 10% buffered formalin, embedded in paraffin, sectioned at 3–5 µm, and stained with haematoxylin and eosin. Histological evaluation was conducted using a scoring system^20^, ^21^, ^22^ to assess inflammation, collagen production and angiogenesis in all the groups; re‐epithelialisation in groups 1 and 2; and epithelialisation in group 3 (Table 1). In group 3, angiogenesis and collagen production were considered only when observed in addition to the pre‐existing tissue. Inflammation and angiogenesis were assessed by counting the total number of inflammatory cells and new capillaries, respectively, in five high‐power fields (400×).

**TABLE 1:** Scoring system for evaluating histological parameters in skin wounds induced in the tarsometatarsus of chickens.

Score	Inflammation	Collagen production	Angiogenesis	Re‐epithelialisation	Epithelialisation
0	Absence (<3 cells)	Absence of collagen fibres	Not observed	Absence (wound completely denuded)	Absence of epithelium (healing by secondary intention)
1	Mild (3–10 cells)	Low amount of collagen fibres	Mild (<3 new capillaries)	Partial (wound partially covered by epithelium)	Partially complete epithelium (with degeneration and necrosis areas)
2	Moderate (11–30 cells)	Moderate amount of collagen fibres	Mild (3‒10 new capillaries)	Complete and continuous (epithelium of reduced thickness compared to the edges)	Intact and thin epithelium (reduced thickness compared to the edges)
3	Severe (>30 cells)	High amount of collagen fibres	Moderate (11‒30 new capillaries)	Complete and continuous (epithelium of similar thickness to the edges)	Complete and continuous (epithelium of similar thickness to the edges)
4	_	_	Marked angiogenesis (>30 new capillaries)	Complete and continuous (epithelium of increased thickness compared to the edges)	Intact and thick epithelium (increased thickness compared to the edges)

### Statistical analysis

Macroscopic data from groups 1 and 2 were tested for normality and homogeneity of variance. The Student's *t‐*test was used for normally distributed data, and the Mann–Whitney test was used for non‐normally distributed data. The percentage of wound area reduction each week (relative to the day of surgery) and weekly wound area measurements were analysed for both groups.

Histological data did not follow a normal distribution. Therefore, the Kruskal–Wallis test was applied to compare groups 1‒3, followed by Dunn's post hoc test for pairwise comparisons. The Mann–Whitney test was used for comparisons between two groups. Statistical significance was set at *p*‐value of less than 0.05. Analyses were performed using SAS 9.3 and Microsoft Excel 365.

## RESULTS

### Macroscopic evaluation

There were no signs of infection during the treatment period. All birds maintained their dressings, and Elizabethan collars were not required. During dressing changes, chickens in group 1 experienced moderate bleeding at the wound centre, whereas no bleeding was observed in groups 2 and 3. In group 1, the wounds exhibited granulation tissue and epithelialisation; however, bone exposure was still present in three chickens on postoperative day 28. By day 28, 71.4% (5/7) of the wounds in group 1 were not fully healed. Group 2 wounds also showed granulation tissue and epithelialisation. Bone exposure was observed in only one chicken on postoperative day 14 and was no longer present at subsequent time points. The L‐TRF membrane appeared thin and dry during dressing changes. By day 28, 87.5% (7/8) of the wounds in group 2 were fully healed. In group 3, two grafts showed signs of necrosis on day 7 post‐surgery. By day 21, four out of eight grafts were fully adhered to the recipient bed. One graft exhibited a blackened spot on day 7, indicating necrosis, but was adhered to the recipient bed by day 28. Another graft appeared pinkish on day 14 but became whitish by days 21 and 28. By day 28, 75% (6/8) of the grafts were viable and 25% (2/8) were necrotic.

**FIGURE 3:**
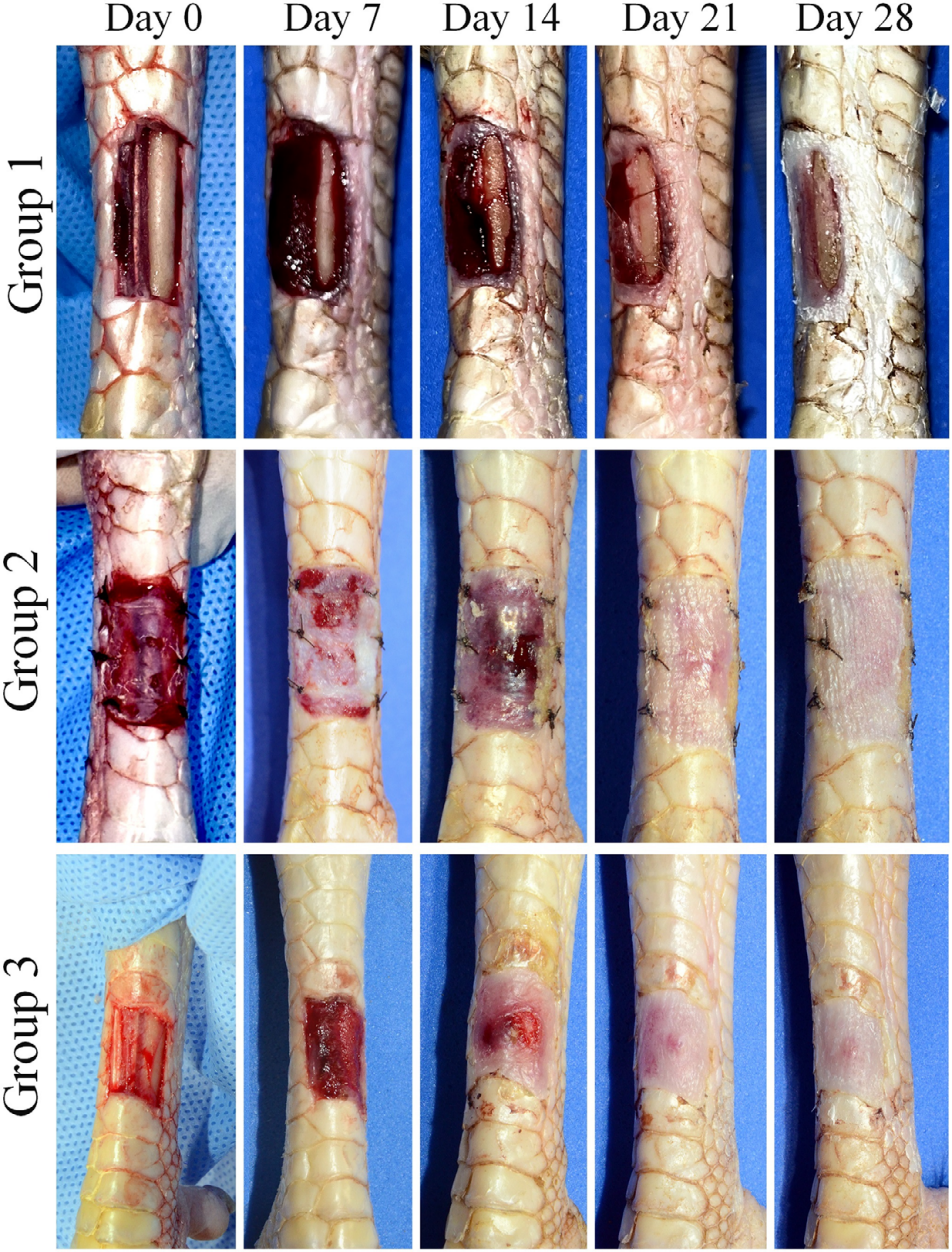
Wounds induced on the tarsometatarsus of chickens in the control group (group 1), treated with a leukocyte‐ and thrombocyte‐rich fibrin membrane (group 2), and with a full‐thickness autologous skin graft (group 3), observed on postoperative days 0, 7, 14, 21 and 28.

Figure 3 shows the macroscopic progression of the skin wounds in groups 1‒3.

### Histological evaluation

**FIGURE 4:**
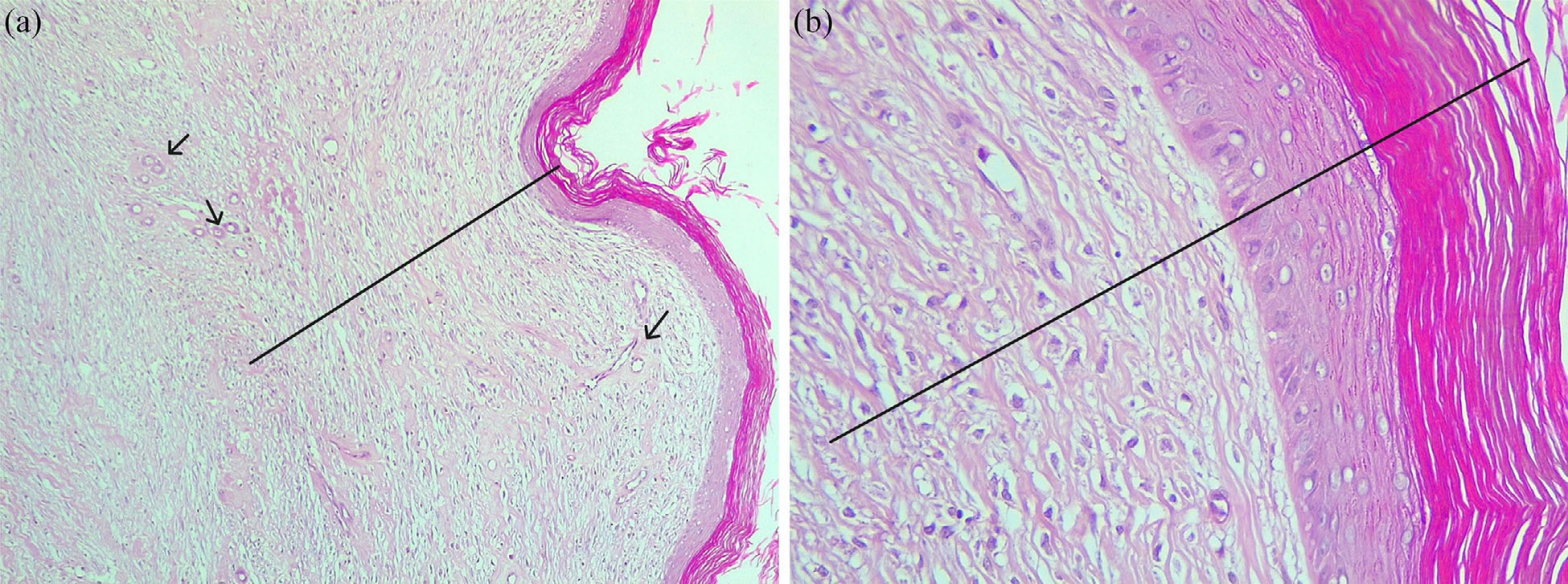
Histological samples of wounds induced on the tarsometatarsus of chickens treated with a leukocyte‐ and thrombocyte‐rich fibrin (L‐TRF) membrane (group 2) on postoperative day 28. (a) Neovascularisation (arrows) and abundant collagen fibres with complete re‐epithelialisation of the wound (straight line) (haematoxylin‒eosin [H&E], magnification ×10). (b) Organised newly formed skin with thick epidermis (straight line) (H&E, magnification ×40).

**FIGURE 5:**
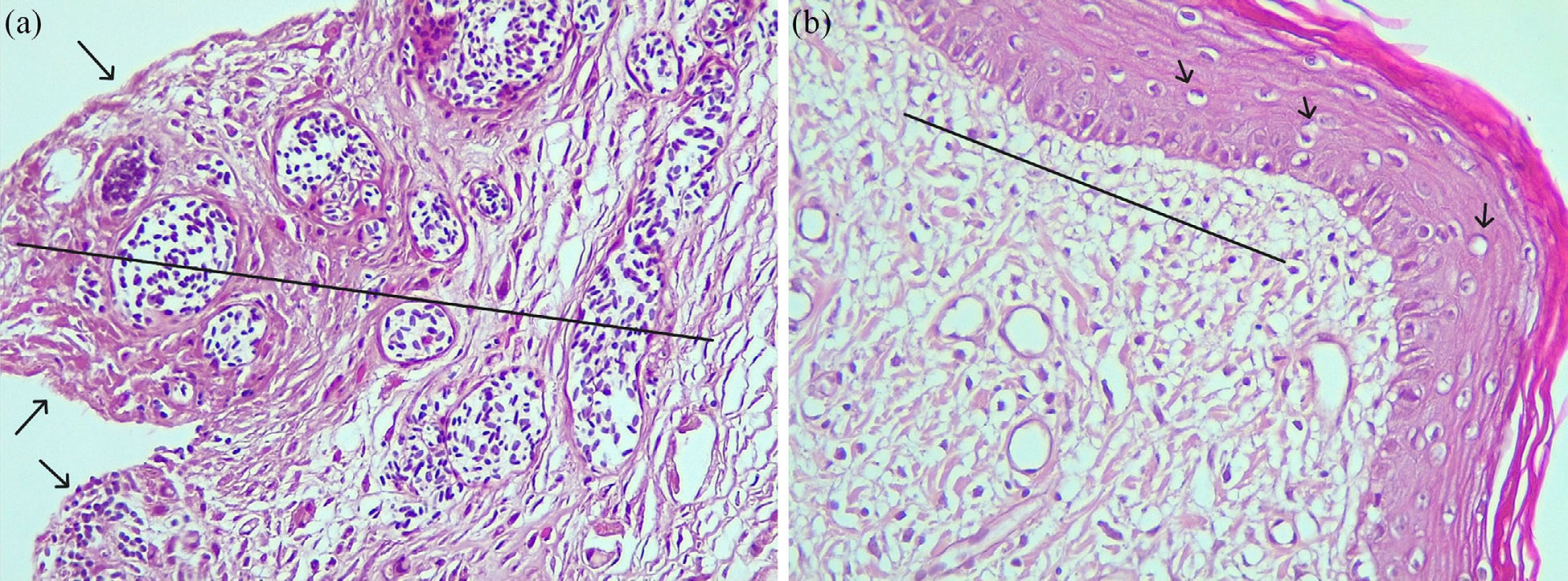
Histological samples of wounds induced on the tarsometatarsus of chickens in the control group (group 1) (a) and those treated with a full‐thickness autologous skin graft (group 3) (b) on postoperative day 28. (a) Area showing absence of re‐epithelialisation (arrows) and a large number of newly formed vessels and collagen fibres (granulation tissue [straight line]). (b) Intact grafted tissue exhibiting moderate oedema (straight line) in the superficial dermis and hydropic degeneration (arrows) of the epidermis, indicating cellular distress (haematoxylin‒eosin, magnification ×40).

In group 1, there was generally considerable proliferation of fibrovascular tissue; however, re‐epithelialisation of the wounds was only partial, originating from the wound edges (Figure 4a). In samples from group 3, epidermal integrity was maintained in most cases (6/8) (Figure 4b). However, oedema was present in both the epidermis (intracellular) and dermis, indicating cellular distress (Figure 4b). In all samples from group 2, organised granulation tissue was observed, along with complete re‐epithelialisation of the wound by an epithelium of increased thickness compared to the wound edges (Figure 5a,b). The inflammatory response in all groups was predominantly composed of lymphocytes and macrophages, with some heterophils also present.

### Statistical analyses

There was a significant difference in the percentage of wound reduction between groups 1 and 2 at all evaluated time points (Table 2). On days 14 and 21, a highly significant reduction was observed. On day 7, there was no statistically significant difference between the two groups in the wound area (Table 3). However, on days 14, 21 and 28, the wound areas differed significantly, with the difference on day 21 being highly significant (Table 3).

**TABLE 2:** Percentage of wound area reduction in skin lesions induced in the tarsometatarsus of chickens from the control group (group 1) and those treated with leukocyte‐ and thrombocyte‐rich fibrin (L‐TRF) membranes (group 2) at 7, 14, 21 and 28 days post‐surgery.

Groups	7 days	14 days	21 days	28 days
1 (control)	20.6 ± 7.1	51.3 ± 16.9	72.3 ± 12.3	90.3 ± 13.0
2 (L‐TRF membrane)	30.6 ± 9.1	84.4 ± 10.8	99.9 ± 3.2	100.0 ± 0.4
*p*‐Values	0.036	<0.001	<0.001	0.029

**TABLE 3:** Area (mm^2^) of skin wounds induced in the tarsometatarsus of chickens from the control group (group 1) and those treated with leukocyte‐ and thrombocyte‐rich fibrin (L‐TRF) membranes (group 2) at 7, 14, 21 and 28 days post‐surgery.

Groups	7 days	14 days	21 days	28 days
1 (control)	121.3 ± 29.5	74.8 ± 35.2	48.5 ± 29.0	13.8 ± 28.2
2 (L‐TRF membrane)	112.9 ± 33.7	25.5 ± 20.1	0.1 ± 4.8	0.0 ± 0.6
*p‐*Values	0.620	0.005	<0.001	0.029

Histopathological evaluation scores for inflammation, collagen production and angiogenesis in the three groups are presented in Table 4. Group 3 showed significantly higher inflammation than group 2. Group 3 showed significantly lower collagen production and angiogenesis compared to the other groups, which did not differ from each other.

**TABLE 4:** Median (interquartile range) of histological evaluation scores for control (group 1), leukocyte‐ and thrombocyte‐rich fibrin (L‐TRF) membrane (group 2) and skin graft (group 3).

Groups	Inflammation	Collagen production	Angiogenesis
1 (control)	2 [2‒2.75] ab	3 [3‒3] a	3 [3‒3.75] a
2 (L‐TRF membrane)	2 [2‒2] b	3 [3‒3] a	3,5 [3‒4] a
3 (skin graft)	3 [2.5‒3] a	2 [1.5‒2] b	2 [2‒2.5] b
*p‐*Values	0.008	<0.001	0.002

*Note*: Medians followed by the same letter in the column do not differ significantly from each other according to Dunn's test (*p* ≥ 0.05).

Regarding epithelialisation in group 3, 25% (*n* = 2) of the wounds had a score of 1, 25% (*n* = 2) had a score of 3, and 50% (*n* = 4) had a score of 4. A highly significant difference in re‐epithelialisation scores was observed between groups 1 and 2, with group 1 showing a median score of 1 and group 2 showing a median score of 4 (Table 5).

**TABLE 5:** Median (interquartile range) of re‐epithelialisation scores for the control (group 1) and the leukocyte‐ and thrombocyte‐rich fibrin (L‐TRF) membrane (group 2).

Groups	Re‐epithelialisation
1 (control)	1 [1‒1]
2 (L‐TRF membrane)	4 [4‒4]
*p*‐Values	<0.001

## DISCUSSION

This study compared two treatment methods for covering large skin wounds with exposed bone in birds. The results revealed differences between the two approaches, highlighting their respective advantages and disadvantages.

When using the L‐TRF membrane in birds, blood volume requirements must be considered. In this study, 2.5 mL of blood (0.18% of bodyweight) was sufficient to produce a membrane covering the defect. While some species (e.g., pigeons, mallards) tolerate blood collection up to 3% of bodyweight,^23^ the general recommendation is to limit it to 1%.^24^ Because serial collections were performed, smaller volumes were used. However, multiple collections may limit applicability depending on species and wound size. The use of homologous or heterologous L‐TRF, as in small animals,^8^ could be an alternative, although its feasibility in birds requires further study due to their thrombocytes replacing platelets.^25^

Although a study using human blood found no differences in PRF membrane structure between dry glass and glass‐coated plastic tubes,^26^ a dry polyethylene terephthalate tube was chosen in this study, as preliminary tests showed it was more suitable for chicken blood. A centrifugation protocol of 1500 rpm for 15 minutes was used, differing from a previous chicken blood study that centrifuged at 3000 rpm for 10 minutes.^13^ Such variations likely stem from differences in centrifuge models, as human blood studies show that vibration profiles vary with speed and can affect L‐PRF clot structure and cell content.^27^ The fibrin clot was converted into a uniform L‐TRF membrane using a PRF Box, a method known to preserve platelet growth factors in human blood.^28^

The percentage of wound reduction was significantly higher in group 2 than in group 1, particularly at 14 and 21 days post‐surgery, likely due to PRF‐derived cytokines such as interleukin‐4 and Vascular Endothelial Growth Factor (VEGF) that promote healing.^6^, ^29^ Fibroblasts and myofibroblasts drive wound contraction during the collagen phase, when epithelial proliferation also occurs.^5^, ^30^ Although a 9.7% greater wound reduction was observed in group 2 compared to group 1 at 28 days, collagen production was histologically similar between the groups. However, unlike group 1, all wounds treated with the L‐TRF membrane achieved complete healing at 28 days postoperatively.

The present study assessed bone exposure as a potential cause of osteomyelitis. In group 2, 87.5% had a wound bed completely filled with granulation tissue 14 days postoperatively, unlike group 1. The PRF membrane protects open wounds, promotes angiogenesis and guides epithelial migration, thereby accelerating healing.^29^, ^31^ However, histological analysis revealed no significant difference in angiogenesis between groups 1 and 2 at 28 days postoperatively. In contrast, according to the re‐epithelialisation score, all wounds in group 2 were fully covered by epithelium, whereas those in group 1 were only partially covered. Considering that the final phase of healing varies with injury severity and persistence,^1^ these findings suggest that wounds treated with the L‐TRF membrane were in a more advanced stage of healing than those in group 1.

Free skin grafts, typically completed in a single procedure, offer an advantage for group 3. However, limited donor sites in birds may pose challenges depending on wound size.^2^ In this study, the inguinal region served as the donor site without complications, consistent with previous reports,^15^ although its thin muscular wall presents potential risks to underlying organs.^2^ A full‐thickness mesh graft was used to facilitate secretion drainage and improve graft–wound contact.^3^ However, bone devoid of connective tissue coverage, as observed in this study, is considered an unfavourable bed for graft acceptance.^14^ Despite this challenge, the decision was made to cover the wound following lesion induction to prevent contamination and to assess the efficacy of skin grafting in avian species.

Group 3 wounds covered with viable skin grafts resembled those in group 2, indicating similar healing progression. Although donor skin contained hair follicles, no feather formation occurred in the recipient bed, consistent with a previous case involving an autologous skin graft used to cover a tarsal lesion in an ostrich.^16^ At the final evaluation, three grafts were dry and non‐adherent, likely due to poor revascularisation resulting from an unfavourable recipient bed, movement or graft thickness. Despite this, they functioned as biological dressings, protecting the wound against adhesions, infection and bleeding.^3^ Because bone without periosteum hinders graft survival, human studies have improved outcomes by drilling the skull's outer table to expose cancellous bone.^32^ This technique may also be considered for birds, but further research is needed to evaluate its effectiveness.

According to the histological analysis, group 3 showed the lowest levels of collagen production and angiogenesis compared to groups 1 and 2. However, 75% of the wounds exhibited an intact epithelium (scores 3 and 4), indicating an advanced stage of healing. This group also differed from group 2 in terms of inflammation, which may be related to the presence of edema in both the epidermis and dermis, along with a predominance of macrophages and lymphocytes—cells typically involved in the early stages of the healing process.^33^

One limitation of the present study is that wound healing in the tarsometatarsus resulted in tissue that differed from the original, regardless of the treatment applied. In chickens, the featherless skin of the tarsometatarsus is covered by overlapping scales (scuta), which consist of a keratinised epidermis.^4^ Therefore, further research is needed to explore alternative strategies for wound treatment in this region.

Based on the macroscopic and histopathological findings, group 2 (L‐TRF membrane) proved to be the most effective treatment for experimentally induced cutaneous wounds on the tarsometatarsus of chickens, compared to group 3 (autologous skin graft).

## AUTHOR CONTRIBUTIONS

Sheila Rahal and Renata Mazaro were responsible for funding acquisition and supervision. Vivian Rech and Sheila Rahal were involved in conceptualisation. Vivian Rech, Luna Rolim, Guilherme Cassanego, Matheus Santos and Rafael Fighera were involved in data collection. Vivian Rech, Sheila Rahal and Renata Mazaro were involved in the data analysis. Vivian Rech and Sheila Rahal curated the data and wrote the original draft, and all the authors contributed to project administration and the review and editing of the paper.

## CONFLICT OF INTEREST STATEMENT

The authors declare no conflicts of interest.

## ETHICS STATEMENT

This study was approved by the Ethics Committee for the Use of Animals (0430/2023‐CEUA) of the School of Veterinary Medicine and Animal Science, UNESP, Botucatu, Brazil.

## Data Availability

The data that support the findings of this study are available from the corresponding author upon reasonable request.
